# Harm Reduction Implications of Vaping Overtaking Smoking in Great Britain

**DOI:** 10.3389/ijph.2026.1609671

**Published:** 2026-04-02

**Authors:** Yusuff Adebayo Adebisi, Riccardo Polosa, Jacob George

**Affiliations:** 1 College of Social Sciences, University of Glasgow, Glasgow, United Kingdom; 2 Nuffield Department of Population Health, University of Oxford, Oxford, United Kingdom; 3 Department of Clinical and Experimental Medicine, University of Catania, Catania, Italy; 4 Cardiovascular Medicine and Therapeutics, School of Medicine, University of Dundee, Dundee, United Kingdom; 5 Medicines and Healthcare products Regulatory Agency (MHRA), London, United Kingdom

**Keywords:** dual use, e-cigarette use, harm reduction, public health, smoking

## Introduction

**FIGURE 1:**
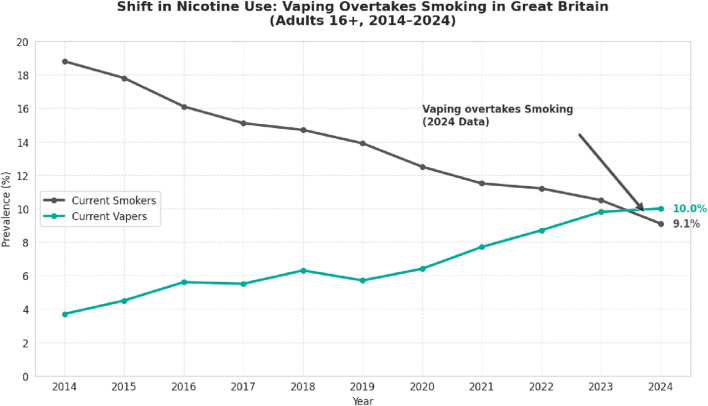
The tipping point: vaping overtakes smoking in Great Britain (2014–2024).

In 2024, for the first time since national records began, more adults in Great Britain used e-cigarettes than smoked tobacco cigarettes [1]. Data released by the UK Office for National Statistics in November 2025 showed that 10.0% of adults aged 16 years and over were current e-cigarette users, compared with 9.1% who smoked tobacco (See Figure 1). This statistical crossover is a notable milestone in tobacco control, occurring alongside sustained declines in smoking prevalence—from 20.2% in 2011 to 10.6% in 2024. While cross-sectional and temporal data limit causal inference, these trends are consistent with harm reduction approaches operating alongside traditional tobacco control measures in driving population-level declines in smoking. 

## User Composition and Patterns of Nicotine Use

To interpret the public health significance of this crossover, it is necessary to examine the composition of e-cigarette users. The ONS reports that 32.8% of current smokers also use e-cigarettes, meaning nearly one-third of smokers are dual users [1]. This equates to approximately 3.0% of all adults. Dual use should be interpreted cautiously; emerging evidence suggests it often represents a transitional stage within a substitution pathway rather than a stable end state for many individuals [2]. Data from Action on Smoking and Health also indicate that among all e-cigarette users, approximately 55% are ex-smokers who have quit smoking completely, 40% are dual users who continue to smoke, and around 5% are never-smokers in Great Britain [3]. This distribution suggests that vaping is concentrated primarily among individuals with a prior smoking history, with complete switching more common than persistent dual use. Supporting this interpretation, ONS estimates show that only 2.7% of never-smokers report vaping [1], indicating that population-level harm reduction benefits likely outweigh risks of nicotine uptake among those unlikely to have smoked otherwise.

## Biomarker Evidence and Toxicant Exposure Reduction

The biological evidence aligns with this risk-continuum interpretation, though important caveats remain. Studies demonstrate that adult smokers who switch completely to electronic nicotine delivery systems experience substantial reductions, often 90% or more, in biomarkers of exposure to tobacco-specific carcinogens such as NNAL and NNN compared with continued smoking [4]. These reductions can approach levels observed in non-users [4]. Dual use should therefore be situated within a gradient of risk rather than framed as outright behavioural failure. For many individuals, dual use is associated with reduced cigarette consumption [5], which may confer meaningful, though incomplete, health benefits relative to unchanged smoking. Recent evidence indicates that dual-use patterns are more behaviourally dynamic than exclusive smoking and frequently progress toward cessation for a proportion of users [2], although persistent dual use does occur and long-term outcomes remain under investigation.

## Smoking Reduction and Dose–Response Health Effects

Beyond biomarker evidence, epidemiological studies provide further insight into the implications of reduced smoking intensity. The relationship between cigarette consumption and smoking-related disease is strongly dose-responsive, though non-linear, with residual risks persisting even at low consumption levels [6]. Recent studies nevertheless demonstrate that incremental reductions in cigarette consumption are associated with lower markers of cardiovascular harm [5]. This supports the potential value of smoking reduction as an intermediate step for smokers unable to achieve immediate abstinence. Given the substantial proportion of smokers who also vape, many of whom report reduced cigarette consumption [2, 5], the aggregate public health impact of reduction pathways warrants continued surveillance and quantification.

## Population Trends and Smoking Cessation Dynamics

At the population level, the temporal association between rising vaping prevalence and sustained declines in smoking prevalence is consistent with vaping contributing to cessation. Although cross-sectional data cannot establish causation, multiple lines of evidence support this interpretation [7]. Smoking prevalence has continued to fall to historic lows during the period in which vaping became widespread [1]. Age-specific trends are particularly instructive in the ONS data: smoking among 18–24-year-olds declined sharply from 25.7% in 2011 to 8.1% in 2024, while vaping prevalence among 16–24-year-olds fell from 15.8% in 2023 to 13.0% in 2024 [1]. This pattern, declining smoking alongside stabilising or declining youth vaping, suggests that any gateway effects are outweighed at the population level by diversion away from combustible tobacco. Comparative observations from Australia and New Zealand further indicate that regulatory environments influencing vaping accessibility may shape smoking-decline trajectories, although such comparisons remain confounded by taxation, health systems, and cultural differences [8].

## Socioeconomic Inequalities in Harm Reduction Uptake

Equitable distribution of potential harm reduction benefits remains a key priority. ONS data confirm socioeconomic gradients in smoking (18.8% in routine/manual occupations vs. 6.5% in managerial/professional) [1]. Accessible alternatives could help address disparities if they reach disadvantaged smokers, who face barriers to traditional cessation. Uptake of disposables among working-age adults (especially 25–49) suggests reach to groups often underserved by other interventions. However, without stratified data on dual use and exclusive vaping by socioeconomic status, it is unclear whether benefits are equitably distributed or if they may accrue disproportionately to certain groups. Further equity-focused research is essential.

## Regulatory Trade-Offs: The Disposable Vape Ban

The UK government’s ban on disposable vapes in June 2025 illustrates the complexity of balancing youth protection with adult harm reduction [9]. Disposable devices have served as a critical entry point for many adult smokers, offering low upfront cost, minimal complexity, and immediate accessibility—factors particularly important for lower-income smokers and those with limited technical literacy or unstable living situations. The policy challenge is to reduce youth access without inadvertently creating barriers that prevent adult smokers from accessing harm reduction tools. Evidence from other jurisdictions demonstrates that overly restrictive regulations can lead to unintended consequences: reduced adult switching, increased smoking relapse among former smokers who vaped, and growth of unregulated markets [8]. A proportionate regulatory approach would maintain youth protections through robust age verification and marketing restrictions while ensuring continued adult access through refillable devices, specialized vape shops, and pharmacy-based distribution.

## Rethinking Tobacco Control Endgame Frameworks

The broader question of tobacco control endgame goals deserves explicit discussion. Traditional frameworks have emphasized complete elimination of tobacco and recently, nicotine use. However, some stakeholders increasingly supports an alternative harm reduction framework that accepts pragmatic endpoints. Complete nicotine abstinence remains the ideal outcome for any individual, but population health is maximized by meeting smokers where they are and offering accessible pathways to reduce harm. The UK’s experience demonstrates that harm reduction and traditional tobacco control can work synergistically rather than in opposition—vaping has expanded during the same period that saw implementation of standardized packaging, comprehensive smoke-free laws, and sustained tobacco taxation increases [10].

## Future Directions for Policy and Surveillance

Important uncertainties remain, including optimising transitions from dual use to cessation, ensuring equitable access, quantifying sustained smoking reduction, and characterising long-term health effects. Nevertheless, current trajectories provide encouraging evidence that substitution pathways can yield measurable population health benefits. Policy priorities should include maintaining proportionate regulation that protects youth while preserving adult access; integrating harm reduction within cessation services; strengthening surveillance systems to monitor behavioural transitions and equity impacts; and avoiding overly restrictive policies that could impede smoking decline. The UK experience offers a potentially instructive model: when smokers are provided with accessible, appealing, and appropriately regulated alternatives to combustible tobacco, substantial public health gains may follow.
